# Primary Choroidal Melanoma in a 30-year-old Woman with Monocular Flashers

**DOI:** 10.5811/cpcem.25339

**Published:** 2025-09-05

**Authors:** Adiba M. Matin, Jacob A. Klinger, Timothy T. Xu, James L. Homme

**Affiliations:** * Mayo Clinic, Department of Emergency Medicine, Rochester, Minnesota; † Mayo Clinic, Department of Ophthalmology, Rochester, Minnesota

**Keywords:** choroidal melanoma, flashers, malignancy, point-of-care ultrasound, uveal

## Abstract

**Case Presentation:**

An otherwise healthy, 30-year-old female was referred to the emergency department by a local optometrist after having flashers and blurry vision for two weeks. Point-of-care ultrasound revealed partial retinal detachment with underlying mass, and dilated fundoscopic examination suggested hyperpigmented lesions. Ophthalmology was consulted, and the diagnosis of amelanotic choroidal melanoma was confirmed.

**Discussion:**

Choroidal melanoma is the most common primary malignant tumor in the eye, but its diagnosis is often delayed due to non-specific symptoms. Early identification is crucial given relatively high rates of metastasis. This case highlights how a tentative diagnosis, made using point-of-care ultrasound and funduscopic examination, can drive timely referral to ophthalmology.

## CASE PRESENTATION

**Image 1:**
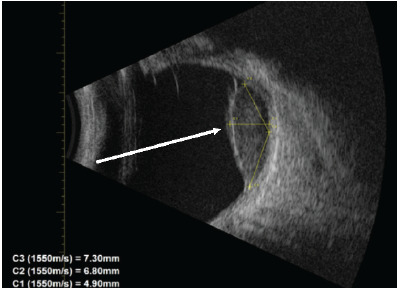
B-scan ultrasound of the left eye demonstrating serous sub-retinal fluid and choroidal mass (arrow).

**Image 2:**
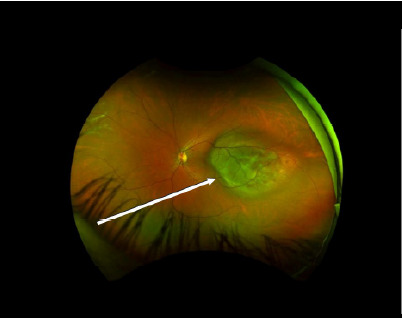
Dilated fundus examination of the left eye showing hyperpigmented lesion (arrow).

**Image 3:**
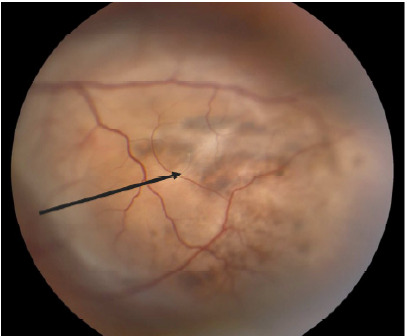
Wide-field fundus image of the left eye demonstrating amelanotic choroidal mass (arrow).

A 30-year-old, otherwise healthy female presented to an optometrist after having “flashers” in the upper quadrant of her left eye for two weeks and blurring of the medial vision of the eye. She had bifrontal headaches for one week. She was referred from there to the emergency department (ED) where a point-of-care ultrasound and dilated funduscopic exam with image acquisition were performed. Point-of-care ultrasound demonstrated a partial retinal detachment with underlying mass (Image 1). Dilated funduscopic exam revealed hyperpigmented lesions (Image 2). Ophthalmology was consulted and evaluated the patient where a diagnosis of an amelanotic left-eye choroidal melanoma was confirmed by non-invasive techniques (Image 3).

She was discharged from the ED in stable condition with expedited follow-up in clinic. Further outpatient evaluation identified pulmonary nodules, a thyroid mass, and liver nodules that were concerning for metastasis. She ultimately underwent fine needle aspiration and plaque radiotherapy.

## DISCUSSION

Ocular and orbital melanomas are rare and life-threatening, malignant etiologies most commonly observed in individuals whose race is White. Uveal melanoma includes the choroid, ciliary body, and iris. However, of these, choroidal melanoma makes up >90% of primary malignant tumors in the eye.^1^ It is the second most common primary tumor seen in malignant melanoma. Most commonly, presentation can be characterized by flashers, floaters, visual field loss, visual acuity loss. However, patients may also present asymptomatic.^2^ Diagnosis is generally made by indirect ophthalmoscopy, A- and B-ultrasonography, fundus fluorescein angiography, and transillumination.^3^

*CPC-EM Capsule*

What do we already know about this clinical entity?

*Choroidal melanoma is the most common ocular malignancy, often associated with visual symptoms, but may also present asymptomatic*.

What is the major impact of the image(s)?

*Point-of-care ultrasound can identify choroidal mass, guiding fundoscopy when available or referral in resource-limited settings*.

How might this improve emergency medicine practice?

*Emergency physicians should consider bedside ultrasound as a readily accessible, low-cost tool for rare but life-threatening conditions like choroidal melanoma*.

Biopsies may not always be required for diagnosis and, as a result, ultrasonography is an important diagnostic tool along with fundoscopy. While ultrasonography is commonly used in diagnosing choroidal melanoma, this case highlights how a tentative, timely diagnosis of choroidal melanoma can be made in the ED using ultrasound and funduscopic examination. The utility of ultrasonography for this purpose in the ED has been under-reported in the literature. Characteristics of a mass that raises concern for melanoma include hollowness and absence of halo.^2^ Size of the mass is the most important prognostic factor for uveal melanoma and predictor of metastasis.^3^ Prompt arrangement of outpatient follow-up with ophthalmology is important in getting a definitive diagnosis and prompt oncologic care.
